# Ultrasonography, computed tomography and magnetic resonance imaging of the dromedary camel distal limbs

**DOI:** 10.1186/s12917-023-03855-2

**Published:** 2024-01-05

**Authors:** Ayman El Nahas, Zakriya Almohamad, Usama Hagag

**Affiliations:** 1 Department of Clinical Sciences, College of Veterinary Medicine, King Faisal University, PO Box 400, 31982 Al-Ahasa, Kingdom of Saudi Arabia; 2 Department of Surgery, Anesthesiology and Radiology, Faculty of Veterinary Medicine, Beni-Suef University, 62511 Beni-Suef, Egypt

**Keywords:** MRI, CT, Ultrasonography, Camel, Distal limbs, Imaging

## Abstract

**Background:**

Lameness associated with the distal limb region in dromedary camels is quiet prevalent. The diagnosis of lameness relies on a comprehensive orthopedic examination conjugated with an appropriate imaging modality to achieve a decisive diagnosis. Using of modern imaging tools provoked a significant breakthrough in the diagnosis of lameness. Ultrasonography (US) is widely established in dromedaries, whereas computed tomography (CT) and magnetic resonance imaging (MRI) are gaining popularity. CT provides a considerably higher bone detail than any other imaging modality. US and MRI continue to be the best options for soft tissue imaging. A truthful assessment of the clinical US, CT and MRI images dictates a comprehensive familiarity with the standard US, CT and MRI tissue deviations. Accordingly, our purposes were to present a full MRI protocol for investigating the dromedary camel distal limbs as well as comparing and illustrating the merits of using MRI, CT and US for evaluation of the front and hind distal limbs in 10 healthy lameness free dromedary camel cadavers. The limbs were scanned via a high-field 1.5 Tesla MRI magnet and a multi-detector CT scanner then subjected to a systematic US examination in both longitudinal and transverse planes. The obtained MRI, CT and US images were evaluated, correlated and compared.

**Results:**

CT and MRI eliminated the structural superimposition in the dromedary camel distal limbs and afforded assessment of minute ligamentous and tendentious structures that were inaccessible by US including the axial collateral ligaments, ligaments supporting the proximal sesamoid bones and the palmar/plantar aspects of the inter-phalangeal joints. US and MRI were appreciated for the assessment of the articular cartilage that was not visible on the plain CT images.

**Conclusions:**

CT and MRI accurately identified and characterized bones and soft tissues constituting the dromedary camel distal limbs. US was appreciated for assessment of soft tissues, articular cartilage and bone contours. CT and MRI may be considered when US results are inconclusive or to evaluate the unreachable parts of the camel distal limbs. Images presented in this study could be used as a reference standard for evaluating dromedary camel distal limb diseases.

## Background

Lameness in the camel distal limbs is a frequent condition that typically results from physical injury or infection. Some distal limb problems involve surgical procedures that require rapid and precise identification of the injury site to reduce patient morbidity [1]. Diagnostic imaging techniques such as ultrasonography (US), computed tomography (CT), and magnetic resonance imaging (MRI) are useful in these cases for exploring and detecting pathological alterations in bones and soft tissues of the distal limbs in camels [2, 3].

Ultrasonography is the preferred modality for outlining soft tissues and bone contours and can offer additional information about the intra-articular structures when combined with radiography [4, 5]. Furthermore, US have been shown to be helpful in the detection of articular cartilage deformities; arthropathies; muscle, tendon and ligament disorders; and neoplastic malformations [6–9]. In particular occasions when radiography or ultrasonography revealed negative outcomes, CT or MRI can provide a distinct judgment [10, 11]. In other circumstances, CT and MRI can be utilized to validate a preliminary diagnosis provided by radiography and/or ultrasonography allowing for further refinement of prognosis and diagnosis [12, 13]. CT has high spatial resolution, providing crisp detail of bony structures in particular [14]. MRI offers a highly detailed examination of bones and soft tissues of the distal limbs [15–17].

The effectual use of US, CT and MRI for investigating the dromedary camel distal limb disorders demands an exhaustive knowledge of the US, CT and MRI features of various soft and osseous structures in limbs without disease [18]. Although the normal US, CT and MRI of the camel limbs are available [19–24], to the best of our knowledge, a standard protocol describing the MRI appearance of the dromedary camel distal limb structures in various sequences is limited and the use of US, CT and MRI for investigation of the dromedary camel distal limbs have not been previously reported together. Consequently, the goals of this work were to describe a reference MRI protocol in multiple sequences for examination of the dromedary camel distal limbs and comparing the obtained MRI images with the US and CT images.

## Results

**Fig. 1:**
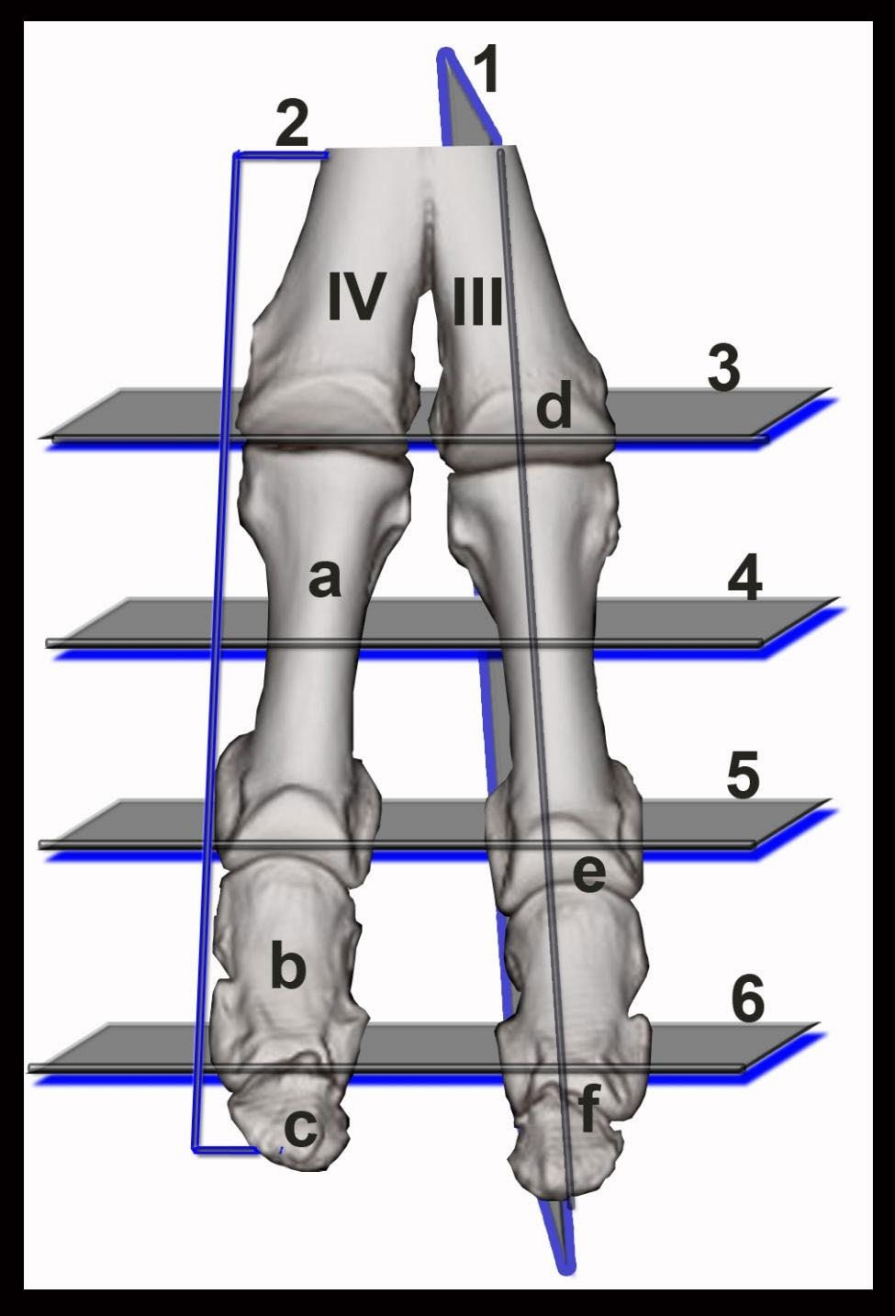
3D CT reconstructed dorsal view of the normal dromedary camel digits. Numbered sections (sagittal, 1; dorsal, 2; and transverse, 3–7) indicate the approximate levels of each computed tomography (CT) and magnetic resonance imaging (MRI) depictions. III, third metacarpal bone; IV, fourth metacarpal bone; a, proximal phalanx; b, middle phalanx; c, distal phalanx; d, fetlock joint;, e, pastern joint; f, coffin joint

**Fig. 2:**
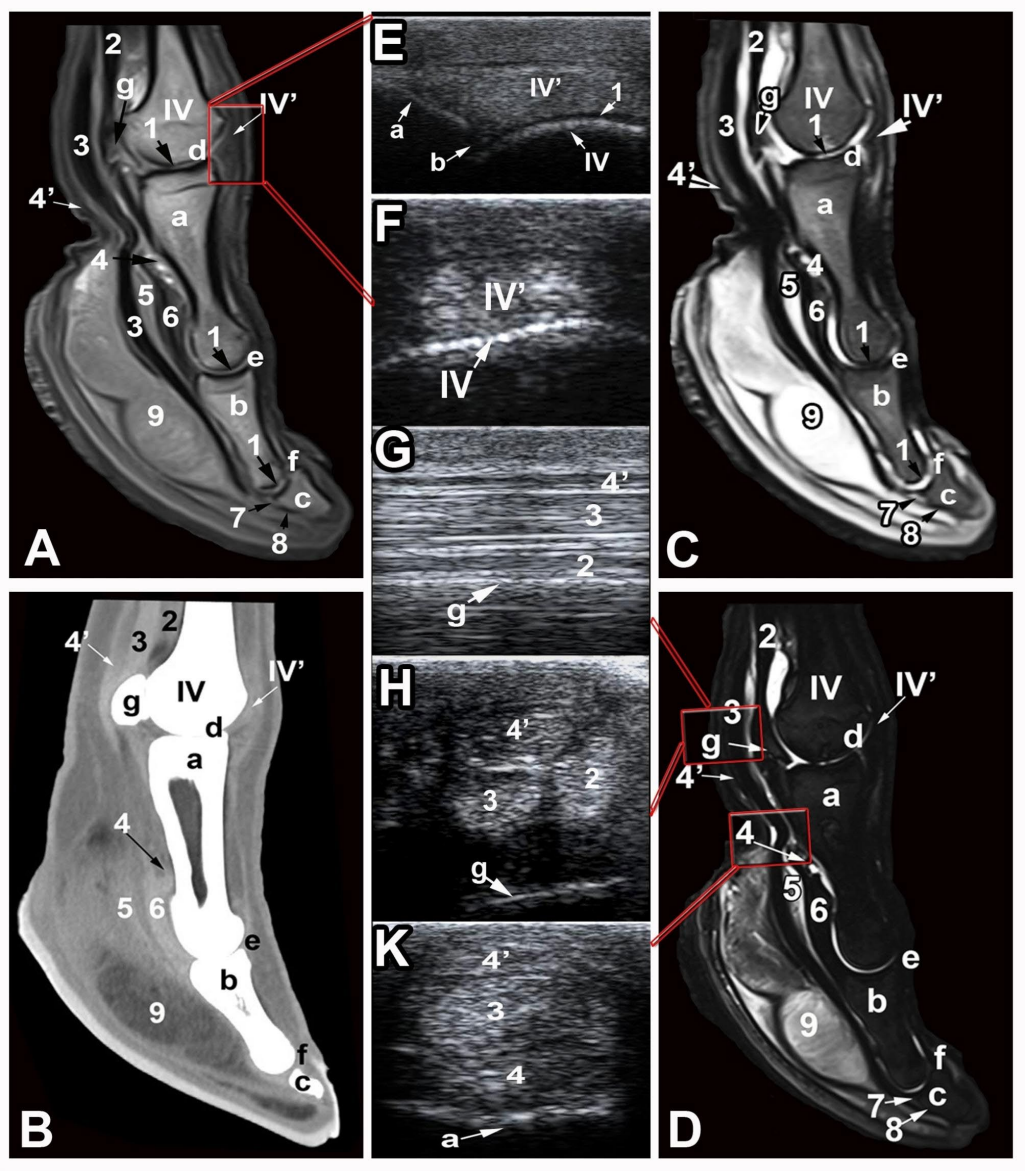
Sagittal T1 (**A**), PD (**C**), and STIR (**D**) weighted MRI images and soft tissue kernel (**B**) CT image of the dromedary camel distal limb (level 1 as indicated in Fig. 1). Longitudinal ultrasound (US) images acquired from a dorsal approach (**E** & **F**) and transverse US images acquired from a palmar/plantar approach (**G, H** & **K**). IV, fourth metacarpal bone; IV’, proper extensor tendon of the fourth digit; a, proximal phalanx; b, middle phalanx; c, distal phalanx; d, fetlock joint;, e, pastern joint; f, coffin joint; g, sesamoid bone; 1, articular cartilage; 2, interosseous medius muscle; 3, DDFT; 4, insertion of the SDFT; 4’, SDFT; 5, fibrocartilagenous enlargement of the DDFT; 6, middle scutum; 7, navicular cartilage; 8, bursa podotrochliaris; 9, adipo-elastic digital cushion

**Fig. 3:**
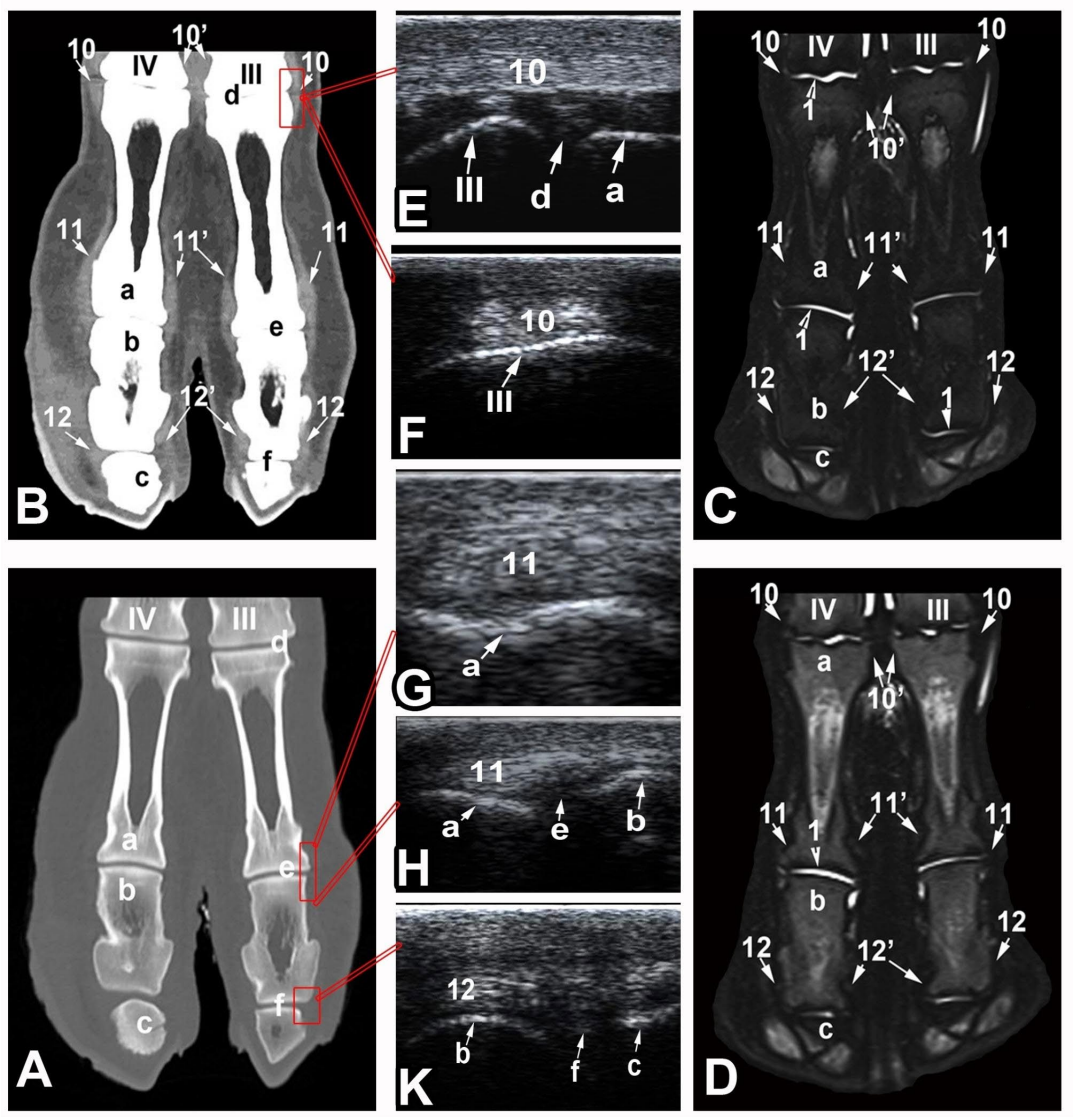
Dorsal bone kernel (**A**) and soft tissue kernel (**B**) CT images and STIR (**C**) and T2 weighted (**D**) MRI images of the dromedary camel distal limb at the level of the collateral ligaments (level 2 as indicated in Fig. 1). Longitudinal ultrasound (US) image (**E, H** & **K**) and transverse (**F** & **G**) US images acquired from an abaxial approach. a, proximal phalanx; b, middle phalanx; c, distal phalanx; d, fetlock joint;, e, pastern joint; f, coffin joint; 1, articular cartilage; 10, abaxial collateral ligament of the fetlock joint; 10’, axial collateral ligament of the fetlock joint; 11, abaxial collateral ligament of the pastern joint; 11’, axial collateral ligament of the pastern joint; 12, abaxial collateral ligament of the coffin joint; 12’, axial collateral ligament of the coffin joint

**Fig. 4:**
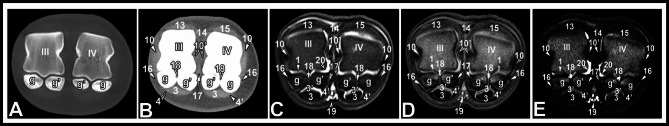
Transverse bone kernel (**A**) and soft tissue kernel (**B**) CT images and PD (**C**), T1 (**D**) and T2 (**E**) MRI images of the dromedary camel distal limb at the level of fetlock joint (level 3 as indicated in Fig. 1). III, third metacarpal bone; IV, fourth metacarpal bone; g, abaxial sesamoid bone; g’, axial sesamoid bone; 1, artricular cartilage; 3, DDFT; 3’, manica flexoria; 4’, SDFT; 10, abaxial collateral ligament of the fetlock joint; 10’, axial collateral ligament of the fetlock joint 13, lateral digital extensor tendon; 14, lateral division of the common digital extensor tendon;15, medial division of the common digital extensor tendon; 16, Collateral sesamoidean ligament; 17, interdigital intersesamoidean ligament; 18, cruciate sesamoidean ligaments; 19, palmar digital artery and vein; 20, synovial fluid of the palmar pouch

**Fig. 5:**
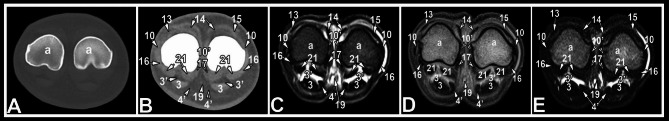
Transverse bone kernel (**A**) and soft tissue kernel (**B**) CT images and PD (**C**), T1 (**D**) and T2 (**E**) MRI images of the dromedary camel distal limb at the middle of the first phalanx (level 4 as indicated in Fig. 1). a, proximal phalanx; 3, deep digital flexor tendon; 3’, manica flexoria; 4’, SDFT; 10, abaxial collateral ligament of the fetlock joint; 10’, axial collateral ligament of the fetlock joint 13, lateral digital extensor tendon; 14, lateral division of the common digital extensor tendon;15, medial division of the common digital extensor tendon; 16, collateral sesamoidean ligament; 17, interdigital intersesamoidean ligament; 19, palmar digital artery and vein; 21, straight sesamoidean ligament

**Fig. 6:**
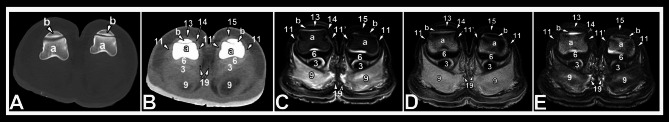
Transverse bone kernel (**A**) and soft tissue kernel (**B**) CT images and PD (**C**), T1 (**D**) and T2 (**E**) MRI images of the dromedary camel distal limb at the level of pastern joint (level 5 as indicated in Fig. 1). a, proximal phalanx; b, middle phalanx; 3, DDFT; 6, middle scutum; 9, adipo-elastic digital cushion; 11, abaxial collateral ligament of the pastern joint; 11’, axial collateral ligament of the pastern joint;axial collateral ligament of the pastern joint; 13, lateral digital extensor tendon; 14, lateral division of the common digital extensor tendon;15, medial division of the common digital extensor tendon; 19, palmar digital artery and vein

**Fig. 7:**
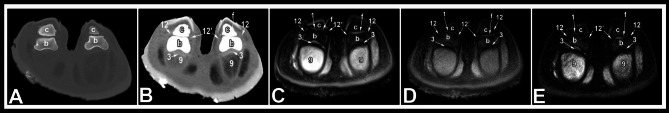
Transverse bone kernel (**A**) and soft tissue kernel (**B**) CT images and PD (**C**), T1 (**D**) and T2 (**E**) MRI images of the dromedary camel distal limb at the level of coffin joint (level 6 as indicated in Fig. 1). b, middle phalanx; c, distal phalanx; f, coffin joint; 1, articular cartilage; 3, DDFT; 9, adipo-elastic digital cushion; 12, abaxial collateral ligament of the coffin joint; 12’; axial collateral ligament of the coffin joint

Six CT and MRI reference figures were selected as being illustrative for the clinically relevant bony and soft tissue structures of the dromedary camel distal limbs (Fig. 1): 1 in sagittal plane (Figs. 2), 1 in dorsal plane (Figs. 3) and 4 in the transverse plane (Figs. 4, 5, 6 and 7). The US images were provided in the longitudinal and transverse planes and were conjugated with their matching CT and MRI images. CT images were furnished in the bone and soft tissue kernels and the MRI images were presented in the T1- weighted turbo spin echo (T1), T2-weighted turbo spin echo (T2), proton density weighted turbo spin echo (PD), and short Tau inversion recovery (STIR) sequences.

The bones constituting the dromedary camel distal limbs involves the distal extremities of the third and fourth metacarpal/metatarsal bones, the proximal sesamoid bones and the duplicated proximal, middle and distal phalanges. The dromedary camel distal limbs involve three couples of joints in each limb: the fetlock, pastern and the coffin joints (Fig. 1). All osseous structures of the distal limbs were visualized and assessed on the CT and MRI images (Figs. 2, 3, 4, 5, 6 and 7). On the US images, the contours of the metacarpal/metatarsal condyles and the dorsal aspects of the proximal, middle and distal phalanges showed hyper-echogenic smooth surfaces with distal acoustic shadowing (Figs. 2 and 3).

The cortical bone showed homogenous hypo-intense signal intensity (SI) and a uniform periosteal and endosteal surfaces on the T1, T2, PD and STIR MRI images, compared with the surrounding tissues. On the US images, the cortical bone had regular margins, whereas on the CT bone window images, it was easily discriminated from the medulla and trabecular bone and possessed a uniform regular endosteal surface. The cancellous bone of the phalanges had homogenous intermediate SI in the T1- and T2-weighted images, except the area of the nutrient vessels that showed increased SI, compared with the cortical bone. The cancellous bone of the distal extremity of the metacarpal/metatarsal bones was well demarcated from the cortical bone and showed homogeneous hypo-intense SI in the STIR images. On the CT bone window, the cancellous bone showed a clearly visible trabecular pattern. The subchondral bone was observed as smooth hyper-echoic lines with concurrent shadowing and reverberation, on the US images; had hypo-intense SI on the T1 – weighted and T2- weighted MRI images and lowered tissue density in the CT bone window images. On the STIR MRI images, the subchondral and trabecular bone had low SI and the subchondral and trabecular bone could not be discriminated and exhibited an appearance of homogeneous hypo-intense SI throughout. On the US images, the articular cartilage of the fetlock, pastern and coffin joints was thin, regular and expressed anechoic appearance. However, US failed to evaluate the navicular cartilage and the deep digital flexor tendon (DDFT) fibrocartilage due to the presence of the fibro-elastic foot pad. On the MRI images, the articular cartilage was seen as a thin layer of homogeneous intermediate to high SI on the T1- weighted images and increased SI on the T2 and STIR images, compared with the surrounding tissues. However, on the CT images, any of the phalangeal articular cartilage or the navicular cartilage could be recognized (Figs. 2 and 3). The articular cartilage over the dorsal surfaces of the proximal sesamoid bones was inaccessible by US and challenging to be evaluated by MRI. The trabecular bone of the proximal sesamoid bones had heterogeneous intermediate to low SI on the MRI images and was clearly differentiated on the CT bone window images. The cortical margins of the proximal sesamoid bones were even, with smooth insertions of the collateral sesamoid and suspensory ligaments on the abaxial aspect and smooth origins of the distal sesamoidean ligaments from the base of each sesamoid bone (Figs. 2 and 3).

The identifiable clinically significant ligaments and tendons of the dromedary camel distal limbs involved the superficial digital flexor tendon (SDFT); DDFT; the common and digital extensor tendons; the suspensory ligament (inter-osseous medius muscle); the axial and abaxial collateral ligaments; sesamoidean ligaments (collateral, cruciate, and the inter-sesamoidean ligaments); the annular ligament; joint capsules; and the foot pad.

The common and lateral digital extensor tendons were seen as uniformly distributed echogenic structures on the US images. The suspensory ligament was detectable as an oval echogenic structure while inserting on the proximal sesamoid bones. The SDFT was more echogenic than the DDFT and the manica flexoria was detected as a strap enclosing the SDFT. The abaxial structures in each of the distal limb joints were unreachable by US. On the CT images adjusted to soft tissue window, the SDFT, DDFT and suspensory ligament were clearly differentiated and smoothly demarcated with high tissue density in relation to the surrounding soft tissues. On MRI images, all the three structures expressed homogeneous low SI on all sequences. The digital flexor sheath could be evaluated on both CT and MRI images (Fig. 3).

On the US images, the abaxial collateral ligaments of the fetlock, pastern and coffin joints could be evaluated and showed an echogenic texture. Ligaments supporting the sesamoid bones were blended to the joint capsule and could not be discriminated. On the CT and MRI images, the collateral ligaments were seen in the transverse and dorsal planes images and expressed high density on the CT soft tissue window images and heterogeneous SI on the MRI images. The ligaments supporting the sesamoid bones including the inter-sesamoidean, collateral sesamoidean and the cruciate ligaments could be detected and showed heterogeneous intermediate SI on all MRI sequences, compared with the surrounding tissues. The short sesamoidean ligaments could not be differentiated (Figs. 4, 5, 6 and 7).

The fetlock, pastern and coffin joints were noticeable on the US images as anechoic gap on the hyper-echogenic bone surface with no detectable synovial fluid inside (Fig. 3). On both the sagittal and dorsal CT slices, the joint spaces were recognizable in the bone window and a hypo-dense synovial fluid was clearly visible. On the MRI images, the joint capsule was clearly outlined by synovial fluid that had intermediate SI on the T1- weighted images and high SI on the T2 – and STIR MRI sequences, compared with the surrounding tissues (Figs. 2 and 3).

## Discussion

The present investigation is the first report presenting and comparing the use of US, CT and MRI together for evaluation of the dromedary camel distal extremities. Furthermore, this is the first study reporting the use of multiple MRI sequences (T1, T2, PD and STIR sequences) for examination of the camel distal limbs. In this study, representative US, CT and MRI images were selected and correlated. Comparing of the US, CT and MRI images together provided a detailed investigation of the soft and osseous tissue structures and allowed a comprehensive characterization of the normal echo-anatomy (US), tissue density (CT) and signal intensity (MRI) of the individual structures constituting the dromedary camel distal limbs. Previous studies in camels reviewed the use of each of the US, CT and MRI for evaluation of some regions in the dromedary camel distal limbs [19–24]. However, in the present study, a systematic US examination of the distal limbs was performed and the validity of the US, CT and MRI techniques for identification of various bony and soft tissues of the dromedary camel distal extremities was evaluated and compared.

The progress of MRI in veterinary practice led to a marvelous advance in the assessment and therapy of musculoskeletal disorders [2]. Typical MRI examination consists of a number of sequences designated by the MRI practitioners. Each MRI individual sequence has its own anatomic and physiologic information, where the appearance of various structures is variable according to the type of the sequence selected. The T1-weighted and PD-weighted sequences display high anatomic information; however, the increased contrast of PD imaging sequence permits a better distinction of anatomic structures compared to the T1 sequence. The PD images compromise a clear distinction between the joint capsule and the synovial fluid that cannot always possible using the T1-weighted sequence. The T2-weighted sequence shows superior contrast and conspicuity of lesions than T1- and PD sequences but lack anatomic detail. The fat suppressed STIR sequence is very informative in detection of unusual fluids in soft tissues and bone containing a considerable amount of fat [25]. Therefore, precise differentiation of certain lesions necessitates the use of multiple sequences in various imaging planes. In previous studies in the camel distal limbs, the use of MRI was confined to a single sequence (T1-weighted sequence). In the present study, an extensive MRI protocol (T1, T2, PD and STIR sequences) for examination of the dromedary camel distal limbs was provided and the appearance of each individual structure on the different MRI sequences was described in order to be used as a reference for evaluation of clinical cases.

Ultrasonography is a noninvasive inexpensive widely available imaging tool that is basically used to evaluate soft tissues and bone surfaces [7]. In the present investigation, systematic examination of the distal metacarpus/metatarsal region and phalanges was presented. US allowed evaluation of the dorsal and abaxial aspects of the fetlock, pastern and coffin joints and was valuable for evaluation of the articular cartilage. However, US lacks completeness, as it was limited to bone surface, the medial (axial) aspect of the joints was unreachable, the palmar/plantar aspects of the pastern and coffin joints were masked by the foot pad and the synovial fluid was undetectable. Similar findings were reported earlier in camel and cattle [3, 20, 21]. On the contrary, CT and MRI were valuable alternatives. The details of osseous structures were more easily identified by CT and MRI and joints and bones constituting the dromedary camel distal extremity were visible in their entirety. CT provided an inclusive assessment of the osseous structures in the dromedary camel distal limbs. The cortical bone, subchondral bone and the trabecular pattern of the cancellous bone was clearly demarcated. However, the articular cartilage and the navicular cartilage were not visible on the plain CT images. In order to assess the articular cartilage, a contrast material is mandatory. On the MRI images, all bones could be evaluated and both the articular cartilage and navicular cartilage were clearly visible.

The use of CT and MRI has significantly improved the diagnostic efficiency for musculoskeletal disorders, compared with radiography and ultrasonography, through minimizing superimposition and increasing tissue detail visualization. CT affords tiny cross sections with high spatial resolution that enables a broad exploration of bone lesions, particularly the alterations in bone density. MRI offers higher soft tissue disparity than CT, which illustrates the degree of pathological variations in both soft and bone tissues. Therefore, CT is regarded as the standard tool for bone pathologies and MRI is the ideal imaging modality for soft tissue disorders [10]. In the present study, US, CT and MRI were of great value for evaluation of soft tissue structures of the dromedary camel distal extremities. However by using of US, the axial collateral ligaments were unreachable; the annular ligament and the digital sheath were undistinguishable; and the ligaments supporting the sesamoid bones were not clearly visible. The clinically important soft tissues in the dromedary distal limbs were recognized and assessed by CT and MRI; however, the spatial resolution of CT images did not exceed the high definition of contrast between tissues generated by MRI.

Results of the current study showed that US, CT and MRI are valuable imaging tools for investigation of the clinically relevant structures in the dromedary camel distal extremities. However, CT and MRI are not screening tools. Efforts should be exerted to define the source of pain causing the lameness via a conventional imaging modality such as ultrasonography before requesting a CT or MRI examination. Information provided in this study is predicted to be helpful in clinical situations.

## Conclusions

Based on the results of this study, we may conclude that US, plain CT and MRI were of great value in the assessment of bones and major soft tissue structures of the dromedary camel distal extremities. Ultrasound is a cost-effective and respectable technique for investigation of soft tissues, articular cartilage and bone surfaces; however, evaluation of the palmar/plantar aspects of the inter-phalangeal joints was limited by the foot pad. CT enabled full assessment of bone tissues and to less extent for soft tissues but plain CT failed to delineate the articular cartilage and navicular cartilage, which requires further contrast CT scanning. The use of different MRI sequences (T1, T2, PD and STIR) was convenient for an inclusive assessment of bones and soft tissues constituting the distal limb of camels. PD-weighted images had the highest contrast that enabled better and easier identification of anatomic structures than T1-weighted images. T2-weighted images were convenient for assessment of synovial structures and can be more helpful in identifying disease processes than T1-weighted images. The STIR sequence reduces the interference from fat signals in the medullary cavity of bones, thereby allowing the identification of medullary fluids in pathological conditions.

## Methods

### Animals

The front and hind limbs of ten adult dromedary camel cadavers were enrolled in this study. Camels were 6 males and 4 non-pregnant females with a mean age of 10 years (range 7–14 years) and a mean weight of 650 kg (range 450–800 kg). Camels were euthanized at the Veterinary Teaching Hospital, College of Veterinary Medicine, King Faisal University for reasons unrelated to the study or orthopedic problems. Immediately after euthanasia, limbs were transected at the carpometacarpal/tarsometatarsal joint and examined fresh within 2 h to avoid imaging artifacts. The lower extremities from the tip of distal phalanx to mid of the metacarpus/metatarsus were studied. Limbs were inspected, palpated and radiographed in dorsopalmar/plantar and lateromedial projections and no abnormal findings were detected.

## MRI protocol

Limbs were positioned with the lateral aspect contacting the MRI table and foot entered the magnet first to replicate clinical positioning. MRI images were acquired using a 1.5 Tesla MRI scanner (Philips Ingenia 1.5T MRI; Philips GmbH, Hamburg, Germany). Limbs were scanned in T1, T2, PD, and STIR sequences in the transverse, sagittal and dorsal planes. Transverse images were acquired perpendicular to the DDFT, while the sagittal images were parallel to the DDFT. The dorsal plane images were perpendicular to the sagittal and transverse planes, aligned with the plane of the metacarpal/metatarsal bone. MRI settings included a repetition time of 800, 2400, 2760 and 2500 milliseconds and echo time of 6, 75, 20 and 16 milliseconds for the T1, T2, PD and STIR sequences, respectively. A slice thickness of 4 mm, gapping of 1 mm, flip angle of 90°, matrix size of 256 and view field of 24 cm, were used.

## CT scanning

Next to the MRI examination, CT scanning was acquired using a multi-detector CT scanner (SOMATOM Force CT scanner; Siemens Healthcare GmbH, Erlangen, Germany). A guide image was created to guarantee symmetry and inclusion of the entire area of interest in the imaging field. Acquisition settings were: 120 kV and 80 mA, slice thickness of 1 mm, inter-slice space of 1 mm, rotation time of 1 s, pitch of 0.63, field of view 20 cm, and matrix size of 512 × 512. Limbs were scanned in a distal-to-proximal direction, from the tip of the distal phalanx to the mid of the metacarpal/metatarsal bone. Transverse images were reconstructed into sagittal and dorsal planes and reviewed by the use of bone settings (window width, 2600 HUs; window level, 350 HUs) and soft tissue settings (window width, 400 HUs; window level, 40 HUs). The density of bones and soft tissues was inspected and recorded.

### Ultrasonographic examination

Finally, Limbs were clipped, rinsed with warm water and soap, dried and covered with a copious amount of the ultrasound coupling gel. The US examination was conducted using B-mode Mindray M5 portable ultrasound machine (Mindray Bio-medical Electronics Co., Ltd, Shenzhen, China) equipped with a 5– 12 MHz multi-frequency linear transducer. The dorsal, abaxial and palmar/planter aspects of the limbs were investigated in both transverse and longitudinal planes at various levels between the proximal and the distal limits of the area of interest and the appropriate US images were selected for further evaluation.

### Image analysis

The obtained US, CT and MRI images were analyzed and representative images were selected. The US images were correlated to their corresponding CT and MRI images. Various osseous and soft tissue structures were identified on each US, CT and MRI images and the echo-pattern, tissue density and signal intensity of each individual structure was described.

## Data Availability

The datasets used and/or analyzed during the current study are available from the corresponding author on reasonable request.

## Abbreviations

MRI: Magnetic resonance imaging
CT: Computed tomography
US: Ultrasonography
TSE: Turbo Spin-echo
T1: T1- weighted turbo spin echo
T2: T2- weighted turbo spin echo
PD: PD- weighted turbo spin echo
STIR: Short Tau inversion recovery
SI: Signal intensity
DDFT: Deep digital flexor tendon
SDFT: Superficial digital flexor tendon
